# Guilt-and Shame-Proneness, Birth-related Post-traumatic Stress and Post-Traumatic Growth in Women with Preterm Birth

**DOI:** 10.1177/00469580241299604

**Published:** 2024-12-02

**Authors:** Gabija Jarašiūnaitė-Fedosejeva, Jovita Kniežaitė, Ernesta Sakalauskienė, Susan Ayers, Annick Bogaerts, Olga Riklikienė

**Affiliations:** 1 Vytautas Magnus University, Lithuania; 2 Lithuanian University of Health Sciences, Lithuania; 3 City, University of London, London, UK; 4 KU Leuven, Leuven, Belgium; 5 University of Antwerp, Antwerp, Belgium; 6 University of Plymouth, Devon, UK

**Keywords:** preterm birth, birth-related post-traumatic stress, post-traumatic growth, guilt-and shame-proneness

## Abstract

Mothers of premature infants are at high risk of experiencing birth trauma and poor postpartum mental health. However, for some, this experience can lead to personal growth. This study examines Lithuanian women with preterm births, where birth-related PTSD is notably higher despite a lower preterm birth rate. Given the common emotional responses of guilt and shame, we explore whether proneness to these emotions moderates the relationship between birth-related PTSD and post-traumatic growth. A cross-sectional study was conducted using an anonymous e-survey to collect data. Women (N = 79) who experienced a preterm birth during 2020 to 2021 participated in the study at least 2 months postpartum, completing the City Birth Trauma Scale (City BiTS), the Guilt and Shame Proneness Scale (GASP), and the Post Traumatic Growth Inventory (PTGI). The relationship between birth-related post-traumatic stress and post-traumatic growth was assessed using linear regression, while the roles of guilt and shame proneness in this relationship were evaluated using moderated regression. The results showed that higher birth-related post-traumatic stress symptoms were associated with greater post-traumatic growth. However, proneness to shame-related negative self-evaluation weakened this relationship, particularly in women with very preterm births. These findings suggest that trauma models should incorporate the moderating role of shame in recovery outcomes. Women with very preterm births who are prone to shame may require more focused attention from healthcare specialists, with targeted interventions to address these emotional challenges and enhance post-traumatic growth. Additionally, policy initiatives should prioritize support programs tailored to the unique psychological needs of these women.

**What do we already know about this topic?**
Women with preterm births often describe them as traumatic, with some studies suggesting these experiences can lead to post-traumatic growth.
**How does your research contribute to the field?**
Proneness to shame weakens the link between birth-related PTSD and post-traumatic growth in women with very preterm births, while guilt does not affect this relationship.
**What are your research’s implications toward theory, practice, or policy?**
Proneness to shame-related negative self-evaluation is important in trauma recovery for women with very preterm births, necessitating that healthcare providers address it and that policies support targeted psychological interventions and early support programs for these mothers.

## Introduction

Preterm birth is defined as babies born alive before 37 weeks of pregnancy, with sub-categories of extremely preterm (less than 28 weeks), very preterm (28 to less than 32 weeks), and moderate to late preterm (32-37 weeks).^1^ Approximately 15 million babies (11% of all births) are born preterm worldwide^2^ and over 5 million in Europe (from 5% to 12% in different countries) each year.^3^ In 2015, the preterm birth rate in Lithuania was lower (5.4%) than in other European countries, while the highest rates were in Cyprus (12%) and Greece (11.3%).^3^ The differences in preterm birth rates could be influenced by a range of factors, including socioeconomic disparities, such as income and education levels, which affect access to quality prenatal care.^4,5^ Variations in healthcare systems, maternal age, and the prevalence of assisted reproductive technologies also play significant roles, with older maternal age and multiple births linked to higher preterm rates.^5^ Cultural and behavioral factors, like smoking and diet, as well as the frequency of medical interventions like cesarean sections, also contribute to these differences.^4^

The number of preterm births is rising in many countries despite medical advances that have reduced mortality for these infants.^3^ The rising numbers of preterm births in high-income countries can be attributed to several factors, including increased maternal age and age-related maternal health issues.^6,7^ The use of assisted reproductive technologies,^8^ which results in an increase in multiple births,^4^ and the higher frequency of medical interventions, such as elective cesarean sections or inductions of labor, may also contribute to the increase in late preterm births (34-36 weeks).^9^ Additionally, the survival of very preterm infants has improved markedly over recent decades due to advances in neonatal care, leading to a higher number of preterm births being recorded in statistics.^10^ In low- and middle-income countries, this could be attributed to maternal health issues such as untreated infections, hypertension, and diabetes, which are exacerbated by limited access to healthcare.^11^

Worldwide, over 1 million children under the age of 5 die every year from complications caused by preterm birth.^12^ Preterm birth remains a significant contributor to infant mortality and morbidity, with far-reaching consequences that can persist throughout a child’s life. These complications can severely impact a child’s neurological development, increasing the risk of cerebral palsy, learning impairments, and visual disorders, as well as long-term disabilities.^13^ Preterm infants also face a heightened risk of chronic health conditions, respiratory and gastrointestinal problems, and developmental delays, all of which can lead to significant physical and educational challenges as they grow.^13^

Mothers of premature children are also at risk for mental health problems^14^ due to the trauma of early delivery, feelings of helplessness, and ongoing anxiety about their child’s health and development, as well as physical exhaustion related to childcare and financial strain from medical expenses associated with the child’s condition and ongoing needs.^15,16^ Studies show, that women with preterm births are at greater risk for the development of postnatal anxiety, depression and post-traumatic stress disorder.^14,17,18^

In many cases, care and support for the preterm newborn are prioritized by health professionals, while the parents’ needs are considered secondary.^19^ A meta-analysis by Citter and Ghanouni^20^ showed that a lack of psychological support for mothers is associated with prolonged psychological difficulties and negative experiences that are harder to resolve. Additionally, missed or delayed care may require long-term additional resources from healthcare specialists, leading to longer maternal recovery times, limited maternal care for the baby, and extended delays in returning to work.^20^

### The Consequences of Traumatic Childbirth Experience

One in three women describes their childbirth as a traumatic experience.^21-23^ The negative effect of a traumatic birth experience is widely recognized, with evidence that traumatic birth experiences can cause tension, negative emotions (anger, fear, anxiety), avoidance behaviors, sleep disturbances and mental health disorders (post-traumatic stress disorder (PTSD), postpartum depression).^24,25^ A recent meta-analysis found that 3% to 6% of mothers experience childbirth-related PTSD,^26^ while the prevalence of subclinical posttraumatic stress symptoms is about twice as high, affecting 12% to 13% of mothers.^26,27^ This prevalence is even higher, particularly among those in high-risk groups like mothers who had preterm births, where 16% to 19% experience subclinical PTSD symptoms.^28^ However, potentially traumatic birth experiences lead not only to negative consequences for the mother but also to positive outcomes such as post-traumatic growth.^29^

Post-traumatic growth has been variously defined. A widely used definition of post-traumatic growth is a positive change in a person’s belief or functioning as a result of the struggle with highly challenging life circumstances.^30^ This includes changes in self-perception (eg, a greater sense of personal strength, improved self-concept), philosophy of life (eg, a greater appreciation for each day, spiritual development), and relationships (eg, deepening of relationships, compassion).^30^ Post-traumatic growth arises as a possible result of trauma and may occur in some individuals but not others.^31^ It is now acknowledged that developmental events, such as childbirth, which are not necessarily traumatic or negative, also have the potential to promote personal growth.^32^ Evidence suggests this is the case for childbirth with up to 50% of women experiencing at least a moderate amount of personal growth following childbirth.^29^ There is also data showing that parents of premature infants experience post-traumatic growth. Wang et al^33^ found that 64.5% of parents who had a pre-term birth experienced moderate to high levels of post-traumatic growth.

### Guilt and Shame Proneness and its Relationship with Traumatic Birth Experiences and Post-Traumatic Growth

Guilt and shame arise from self-reflection when a person fails to meet certain standards of social and moral behavior, serving as immediate emotional responses that reinforce or punish behavior, thereby acting as a moral compass.^34^ Although they often occur together, they are distinct emotions with different focuses.^35,36^ Shame is described as a self-conscious emotion triggered by a negative evaluation of oneself, often involving feelings of worthlessness or inadequacy. It reflects a sense of failing to live up to societal or personal standards, focusing on the self rather than specific behaviors.^34-36^ In contrast, guilt is defined as a self-conscious emotion that arises when one perceives a specific behavior as wrong or harmful.^34-36^ Unlike shame, guilt is more focused on the behavior itself rather than on one’s overall self-worth and often leads to feelings of responsibility and a desire to make amends. Guilt and shame proneness is a personality trait that reflects a person’s general tendency to experience guilt and shame more readily and frequently than others.^34^ To be more specific, guilt proneness is the tendency to feel bad about specific actions, leading to a focus on making amends or repairing situations or damage. Shame proneness, on the other hand, is the tendency to feel bad about oneself, leading to feelings of inadequacy and a tendency to withdraw.^37^ When measuring guilt and shame proneness, both feelings and action tendencies are usually assessed.

Guilt and shame are emotions commonly associated with motherhood. According to self-discrepancy theory, these emotions arise from perceived gaps between one’s actual and ideal selves.^38^ Parents who are prone to guilt may constantly worry about whether they are doing enough or making the right decisions for their child.^38,39^ They might feel responsible for every negative outcome, even when it’s beyond their control, leading to overcompensation, anxiety, or excessive self-criticism.^39,40^ Shame-prone parents might struggle with feelings of inadequacy and fear of being judged by others, leading them to hide perceived shortcomings of parenting or avoid seeking help.^39^ This can result in feelings of isolation, reluctance to share parenting struggles, or an overly harsh self-assessment.^38,39^

In the context of childbirth, women may experience guilt and shame when their birth experience does not align with their expectations. For example, a woman might feel guilty after complications during pregnancy or childbirth, such as preterm birth or cesarean section, labor augmentation, or choosing pain relief, thinking, “I didn’t manage to give birth normally/without help,” believing they could have prevented these outcomes.^41^ Shame may manifest as a deep sense of personal failure if the birth does not go as planned (eg, needing a C-section instead of a natural birth). This can lead to feelings of embarrassment or inferiority compared to other mothers, potentially affecting their self-esteem and mental well-being.^41^ Studies have shown that negative birth experiences can predict feelings of shame,^42^ and women may also feel guilt for outcomes related to pregnancy or birth, such as miscarriage.^43^ When discussing the context of pre-term birth, Barr^44^ suggests that mothers may experience self-blame that can be either shame-based or guilt-based. This self-blame can manifest as shame, where they see themselves as fundamentally flawed, or as guilt, where they regret specific actions or inactions.

There are no studies directly analyzing the relationship between guilt and shame proneness and PTSD symptoms or post-traumatic growth in the perinatal or postnatal context. However, one study^45^ showed that guilt and shame proneness were associated with longer and more intense grief following perinatal bereavement in both women and men. Since perinatal death of a newborn is a traumatic event, it is reasonable to assume that similar emotional responses could contribute to the development and persistence of birth-related PTSD, with these self-conscious emotions potentially exacerbating the psychological impact of a traumatic experience.

The relationship between guilt and shame proneness with PTSD symptoms and post-traumatic growth has been explored in other contexts involving trauma survivors. Shi et al^46^ found that both shame and guilt are positively associated with PTSD symptoms, suggesting that these self-conscious emotions may similarly contribute to PTSD development. Findings from a random-effects meta-analysis further support the significance of both emotions in their association with PTSD.^47,48^

Interestingly, some studies highlight that proneness to shame may have a stronger relationship with PTSD symptoms than guilt,^49,50^ particularly in the maintenance of these symptoms over time. This raises the question of whether shame, which is more self-focused, might have a more pervasive impact on PTSD by undermining a person’s sense of self-worth and social connection.

In contrast, Bub and Lommen^51^ demonstrated that guilt, while contributing to PTSD symptomatology, might be part of a causal mechanism, possibly because guilt is more behavior-focused and could drive rumination on specific actions. However, Cunningham et al^52^ found that when both emotions were analyzed together, shame became the dominant predictor, rendering guilt’s impact non-significant. This finding underscores the potentially more damaging role of shame in PTSD, as it may encompass a broader, more debilitating self-assessment than guilt.

Kleim and Ehlers^53^ found that feelings of shame predicted post-traumatic growth. However, in Kopecki’s^54^ study, only guilt proneness significantly predicted post-traumatic growth, while shame proneness did not make a unique contribution to post-traumatic growth.

Post-traumatic growth is a consequence of a traumatic experience.^55^ In contrast, proneness to guilt and shame is considered a stable personality trait,^34^ making it unlikely to change during pregnancy or postpartum. Proneness to shame and guilt is associated with PTSD symptoms and post-traumatic growth, suggesting that these self-conscious emotions may play a role in influencing these outcomes. It is therefore possible that proneness to guilt and shame affects the relationship between traumatic birth experiences and post-traumatic growth. Both guilt and shame can lead mothers to deeply reflect on their experiences, driving them to understand and make sense of what happened. However, due to differences between these emotions, proneness to guilt can motivate mothers to make amends and improve themselves. This desire for positive change can foster behaviors and thought patterns that facilitate post-traumatic growth, such as seeking social support, learning from the experience, and adopting new life priorities. In contrast, shame might lead to excessive rumination, self-blame, and a negative self-image. These emotions can exacerbate PTSD symptoms and hinder the healing process. Instead of facilitating growth, they can trap the individual in a cycle of distress and self-criticism, making it harder to achieve post-traumatic growth. Shame can also lead to avoidance behaviors and social withdrawal, preventing individuals from seeking support or engaging in the cognitive processing necessary for post-traumatic growth. This can weaken the relationship between birth-related PTSD and post-traumatic growth, as the individual may become stuck in their trauma without finding a path to recovery.

Women who have had preterm births are at high risk of experiencing their birth as traumatic, often leading to feelings of guilt and shame for not being able to carry their baby to full term.^56^ Although the prevalence of preterm births among Lithuanian women is relatively low compared to other European countries, the incidence of childbirth-related PTSD is notably higher, at 7.97%.^57^ Given these factors, it is crucial to explore how Lithuanian women who have had preterm births navigate their trauma and potentially experience posttraumatic growth. Understanding the relationship between birth-related PTSD, posttraumatic growth, and the effects of shame and guilt proneness in this specific population can provide valuable insights into culturally sensitive resilience and recovery processes.

Thus, the aim of this study was to explore the moderating effect of proneness to guilt and shame on the relationship between birth-related post-traumatic stress and post-traumatic growth in women with preterm births. Based on the evidence, it was hypothesized that women who had preterm births that they perceived as traumatic would experience greater post-traumatic growth. Additionally, it was hypothesized that proneness to guilt would strengthen the relationship between symptoms of birth-related PTSD and post-traumatic growth, while proneness to shame would weaken this relationship.

## Method

### Design and Method

A quantitative cross-sectional study conducted using an anonymous e-survey.

### Participants

Before data collection, the required sample size was calculated. Several rules of thumb, such as the sample-to-variable ratio suggested by Tabachnick and Fidell,^58^ were considered, which proposes 15 to 20 observations per independent variable. In an analysis with 3 predictors, this would suggest a sample size of 45 to 60. Additionally, a popular rule of thumb by Jackson^59^ was referenced, which recommends a ratio of observations to predictors, where a ratio of 20:1 is ideal, 10:1 is good, and 5:1 is minimal. Based on this guideline, this study would require a sample size of 30 to 60 for a good or ideal sample size.

Finally, the G*Power program^60^ was used to calculate the a priori sample size, assuming an effect size of 0.15, an α error probability of .05, and a power (1-β error probability) of .8, which are commonly used in social science research, as suggested by Memon et al^61^ for a moderated regression model with 3 predictors. The total sample size based on these conditions was calculated to be 77. Therefore, the goal was to collect a sample of at least 77 women who had experienced pre-term birth.

In total, 79 Lithuanian women who had a preterm birth during 2020 to 2021 participated in a study. Eligibility criteria were that women had to have experienced preterm birth (gestational age of 36 weeks or less) at least 2 months ago, and a maximum of 14 months ago. The study involved women 2 to 14 months postpartum (mean 5.95, SD 3.82). Age of participants varied from 21 to 44 years (mean 31.42, SD 5.22). The gestational age of the newborn ranged from 22 to 36 weeks (mean 31.44, SD 3.98). The majority of women (70.9%) had attended higher education and were married or lived with a partner (95%). The number of previous childbirths varied from 1 to 7 (median 2, mode 1), and the number of children women had ranged from 1 to 8 (median 2, mode 1). In regard to the health status of the newborn, 74.7% of women indicated having healthy babies, and 19.0% responded having babies with prematurity related illnesses or health problems (6.3% did not provide an answer to this question).

### Measures

All the scales used in this study were backwards translated into Lithuanian following accepted methodological requirements.^62^

The City Birth Trauma Scale (City BiTS) was used for measuring the symptoms of childbirth-related post-traumatic stress.^63^ The scale consists of 29 items that comprise four subscales: re-experiencing symptoms, avoidance symptoms, negative cognitions and mood, hyperarousal. Participants were asked to report the frequency of these symptoms experienced over the past week, with scores ranging from 0 (“not at all”) to 3 (“5 or more times”). An example of an item is: “Recurrent unwanted memories of the birth (or parts of the birth) that you can’t control.” A higher score on a scale indicates greater severity of symptoms of postpartum PTSD. The scale’s internal consistency is good (Cronbach’s alpha .92).^63^ Cronbach’s alpha in this sample was similar (α = .88).

The Guilt and Shame Proneness Scale (GASP) was used to measure proneness to guilt and shame.^37^ The scale consists of scenarios that people are likely to encounter in day-to-day life, followed by common reactions to those situations. Respondents are asked to read each scenario, imagine themselves in that situation, and indicate the likelihood that they would react in the described way using a 1 to 7 Likert scale, where 1 indicates “very unlikely” and 7 indicates “very likely.” An example of a scenario is: “After realizing you have received too much change at a store, you decide to keep it because the salesclerk doesn’t notice. What is the likelihood that you would feel uncomfortable about keeping the money?” The scale consists of four subscales, two of which measure proneness to guilt (negative behavior evaluation and repair action tendencies); and two of which measure proneness to shame (shame-related negative self-evaluation and withdrawal action tendencies). So, the scale measures not only proneness to guilt- and shame-related feelings but also associated action tendencies. A higher score on each subscale indicates greater proneness to guilt- or shame-related feelings or action tendencies. The authors suggest examining the effects of each subscale separately in multiple regression analysis to avoid multi-collinearity. Internal consistency of subscales indicates Cronbach’s alpha above .60.^37^ Internal consistency of the subscales in the sample ranged from α = .62 to .81.

The Post Traumatic Growth Scale (PTGI) was used to measure post-traumatic growth.^64^ The respondents were asked to report the degree of change they experienced in their lives as a result of pre-term birth using a 0 to 5 Likert scale, where 0 indicates “I did not experience this change as a result of pre-term birth” and 5 indicates “I experienced this change to a very great degree as a result of having a pre-term birth.” An example of an item on the scale is: “I have a greater feeling of self-reliance.” The scale consists of 21 items that comprise five subscales: relating to others, new possibilities, personal strength, spiritual change, and appreciation of life. A higher score on a scale indicates greater post-traumatic growth. The scale’s internal consistency is good (Cronbach’s alpha .90).^64^ Cronbach’s alpha in this study was α = .95.

Sociodemographic and birth-related questions were included to measure participants age, education, marital status, number of children in a family, time since childbirth, gestational age of the newborn (duration of pregnancy), number of births women have had, and the health status of the newborn as perceived subjectively by the mother (healthy or unhealthy).

### Procedure

The Regional Committee on the Biomedical Research Ethics granted the permission to conduct the study (No. BE-2-73). Invitations to participate in the study were posted on Facebook groups for mothers who have experienced preterm birth (eg, *Pre-term babies*, *Pre-term babies and mothers’ fears*), and in groups for mothers who had similar times of childbirth (eg, *Mothers of 2020/2021*, “*Babies of July 2020*,” etc.). Some of the maternity wards at university, region or district health care institutions and the organization “*Helping to grow*” also disseminated the invitation to participate on their Facebook pages. In addition, women visiting the outpatient neonatal unit of the obstetric clinics at a university hospital were informed about the study. The survey was available to complete online via LimeSurvey software platform.

When opening the link to the questionnaire, participants were provided with information about the aims of the study, research team, inclusion criteria of study participants, potential harm and benefits of participating in a study, rights of participants, information on anonymity and confidentiality, and the contact details of the researchers in case they had any questions. After this, participants were asked to check a box to indicate their consent to participate. At the end of the survey, respondents were thanked for participation and provided with contacts of emotional helplines, if necessary. Participation was voluntary and participants were not given any money or gifts for participating. The data was collected from January to August 2021.

### Statistical Approach

The data were analyzed using IBM Statistical Package for Social Sciences (SPSS 23) and Mplus7. The relationships between birth-related post-traumatic stress, post-traumatic growth, proneness to shame and guilt, and sociodemographic characteristics such as age, number of children in a family as well as childbirth-related indicators such as time after childbirth, gestational age (duration of the pregnancy), and number of births were evaluated using Pearson or Spearman’s correlations (depending on satisfaction of the assumption of normality of the data and the level of measurement of the variables). Differences in study variables depending on women’s education and newborn’s health were evaluated using the independent samples Student *t*-test. Means, standard deviations, and bivariate correlations (*r*) of the main study variables (birth-related post-traumatic stress, post-traumatic growth, proneness to shame and guilt) were calculated as well.

Linear regression analysis was used to test the hypothesis that women who experienced preterm birth as more traumatic (have more symptoms of postpartum PTSD) experience greater post-traumatic growth. Four moderated regression models were used to test the hypothesis that proneness to guilt and shame enhance the association between birth-related traumatic experiences and post-traumatic growth in women who have experienced preterm birth. The sub-scales of proneness to guilt and shame were placed separately in the individual models to avoid multicollinearity. *R*² change (extra variance explained) was calculated to get a moderation effect and an effect size. To explore the nature of the interactions, simple slope tests were calculated; significant interactions were also plotted. About 10.000 bootstrap samples and a confidence interval of 95% were selected for analysis.

The significant moderated regression models were also analyzed in two gestational age groups: very preterm (up to 32 weeks) and moderate to late preterm (32-36 weeks). Significance in the entire analysis was defined by a *P*-value of .05.

## Results

Before testing the hypotheses, the relationship between the studied phenomena and sociodemographic and childbirth-related indicators was evaluated (see Supplemental Material Table S1 and Table S2). A statistically significant negative relationship was observed between duration of pregnancy and post-traumatic growth (r = −.323; *P* = .004) with post-traumatic growth increasing with lower gestational age. There was a significant relationship between a newborn’s health status and symptoms of birth-related PTSD (*t* = 3.485, df = 72, *P* = .001) where women whose newborn was not healthy experiencing more symptoms of birth-related PTSD than those with healthy babies.

Means, standard deviations, and bivariate correlations (*r*) of the main study variables were also calculated and are presented in Table 1.

**Table 1.** Descriptive Statistics and Correlations Among Study Variables.

Variables	Range (min-max)	*M* (SD)	1	2	3	4	5
1. Birth-related PTSD symptoms	0-40	14.48 (10.15)	1				
2. Post-traumatic growth	0-99	48.62 (25.15)	0.316*	1			
3. Guilt-related negative behavior evaluation	6-28	22.62 (5.51)	−0.022	−0.055	1		
4. Guilt-related repair action tendencies	4-28	21.76 (5.35)	0.091	0.077	0.741**	1	
5. Shame-related negative self-evaluation	5-28	21.44 (4.85)	0.257*	0.271*	0.449**	0.473**	1
6. Shame-related withdrawal tendencies	4-26	12.62 (5.29)	0.136	0.095	0.168	0.100	0.282*

Pearson correlation, *P < .05; **P < .01.

The linear regression to assess the relationship between the severity of birth-related PTSD symptoms and post-traumatic growth, taking into account the duration of pregnancy and the health of the newborn is shown in Table 2.

**Table 2.** The Relationship Between Birth-related PTSD symptoms and Post-traumatic Growth.

Predictors and covariates	*B*	Std. error	β	*t*	*P-*value	95% CI
Birth-related PTSD symptoms	0.739	0.289	.297	2.554	.013	[0.162; 1.315
Gestational age	−1.986	0.702	−.308	−2.829	.006	[−3.386; −0.586]
Heath of a newborn	1.526	7.275	.025	0.210	.834	[−12.983; 16.036]
*R*²	.187					

*F* (73) = 5.384, *P* = .002.

The results showed that post-traumatic growth was significantly predicted by birth-related PTSD symptoms (β = .297; *P* = .013) and gestational age (β = −.308; *P* = .006). Once these factors were taken into account the health of the newborn was not related to post-traumatic growth (*P* = .834). This supports the hypothesis that women with preterm birth who experienced their childbirth as more traumatic also experienced higher post-traumatic growth.

Four moderation models separately assessed the effect of guilt and shame proneness subscales on the relationship between birth-related PTSD symptoms and post-traumatic growth. The results indicated that only the moderated regression model using proneness to shame-related negative self-evaluation as a moderator was significant, while other potential moderators, such as proneness to shame-related withdrawal tendencies, guilt-related negative behavior evaluation, and guilt-related repair tendencies, did not show a significant effect (Table 3).

**Table 3.** The Effect of Shame and Guilt Proneness on the Relationship Between Birth-related PTSD symptoms and Post-traumatic Growth.

Predictors, moderators and interaction terms	*B*	Est./S.E.	*P*-value	95% CI
Model 1				
Symptoms of birth-related PTSD	3.109	2.940	.003	1.370; 4.849
Shame-related negative self-evaluation	2.663	2.744	.006	[1.066; 4.260]
Birth-related PTSD symptoms × shame-related negative self-evaluation	−0.114	−2.341	.019	[−0.194; −0.034]
*R*²	.178			
Model 2				
Symptoms of birth-related PTSD	1.112	0.689	.111	[−0.260; 2.484]
Shame-related withdrawal tendencies	1.129	1.050	.286	[−0.963; 3.222]
Birth-related PTSD symptoms × shame-related withdrawal tendencies	−0.039	0.053	.463	[−0.144; 0.066]
*R*²	.091			
Model 3				
Symptoms of birth-related PTSD	2.186	1.059	.042	[0.077; 4.295]
Guilt-related negative behavior evaluation	1.042	0.792	.192	[−0.535; 2.620]
Birth-related PTSD symptoms × guilt-related negative-behavior-evaluation	−0.069	0.047	.145	[−0.162; 0.024]
*R*²	.102			
Model 4				
Symptoms of birth-related PTSD	1.844	1.093	.096	[−0.334; 4.021]
Guilt-related repair action tendencies	1.345	0.967	.168	[−0.580; 3.270]
Birth-related PTSD symptoms × guilt-related repair action tendencies	−0.054	0.049	.273	[−0.152; 0.043]
*R*²	.098			

As shown in Table 3, all variables in the model were significantly associated with post-traumatic growth: birth-related PTSD symptoms (*B* = 3.266, *P* = .004), proneness to shame-related negative self-evaluation (*B* = 2.949, *P* = .005) and the interaction between traumatic childbirth experience and proneness to shame-related negative self-evaluation (*B* = −0.125, *P* = .016). Thus, proneness to shame-related negative self-evaluation moderated the relationship between birth-related PTSD symptoms and post-traumatic growth. Higher levels of birth-related PTSD symptoms were associated with greater post-traumatic growth, and a greater proneness to shame-related negative self-evaluation was also related to higher post-traumatic growth. However, in assessing the moderating effect, an increase in proneness to shame-related negative self-evaluation decreased the effect of traumatic birth experience on post-traumatic growth. Thus, proneness to shame-related negative self-evaluation weakened the relationship between birth-related PTSD symptoms and post-traumatic growth.

**Figure 1.**
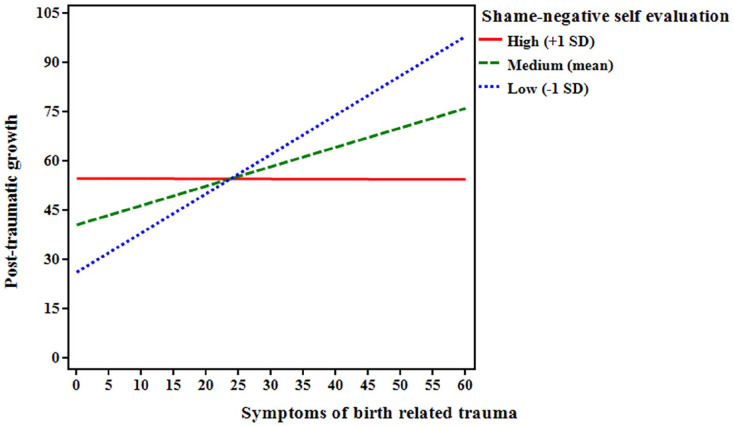
The effect of proneness to shame related negative self-evaluation of the relationship between birth-related trauma symptoms and post-traumatic growth.

To explore the nature of the interaction, a simple slope test was calculated between three levels of the moderator: high proneness to shame-related negative self-evaluation (+1SD), medium proneness to shame-related negative self-evaluation (mean), and low proneness to shame-related negative self-evaluation (−1SD). Simple slope analysis revealed that even though proneness to shame-related negative self-evaluation was positively related to post-traumatic growth (as shown by the moderation model), this relationship was stronger for lower (*B* = 3.223, *P* = .004) and medium (*B* = 3.109, *P* = .003) proneness to shame-related negative self-evaluation than it was for high proneness to shame-related negative self-evaluation (*B* = 2.995, *P* = .003) (see Figure 1).

Because gestational age was significantly related to post-traumatic growth, the model was repeated with gestational age included. In this model, the interaction term was not significant, meaning that proneness to shame-related negative self-evaluation did not moderate the relationship between birth-related trauma and post-traumatic growth once gestational age was controlled for (*P* > .05).

To find out why the inclusion of gestational age as a covariate in moderation analysis made the moderation effect non-significant, additional calculations were performed on that including two groups based on gestational age¹: very preterm (up to 32 weeks, N = 35) and moderate to late preterm birth (32-36 weeks, N = 44). The results revealed that the moderator effect was statistically significant only in the very preterm birth group. In contrast, the moderator effect was not statistically significant in the moderate to the late preterm birth group.

**Figure 2.**
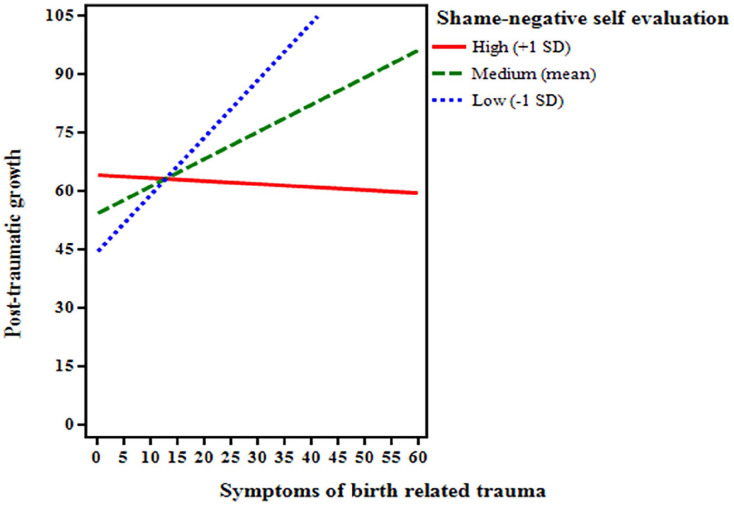
The effect of negative self-evaluation associated with a proneness to shame on the relationship between birth-related trauma symptoms and post-traumatic growth in the very preterm birth group.

The effect of proneness to shame-related negative self-evaluation for the relationship between birth-related trauma experience and post-traumatic growth in the very preterm birth group is presented in Table 4 and Figure 2.

**Table 4.** The Effect of Negative Self-evaluation Associated With a Proneness to Shame on the Relationship Between Birth-related PTSD symptoms and Post-traumatic Growth in the Very Preterm Birth Group.

Predictor, moderator and interaction term	B	Est./S.E.	*P-*value	95% CI
Symptoms of birth-related PTSD	3.312	1.320	.012	[1.141; 5.900
Shame-related negative self-evaluation	18.034	7.404	.015	[5.854; 30.214]
Birth-related PTSD symptoms × shame-related negative self-evaluation	−0.121	0.065	.049	[−0.227; −0.014]
*R*²	.256			

As shown in Table 4, all variables in the model were significantly associated with post-traumatic growth: birth-related PTSD symptoms (*B* = 3.312, *P* = .012), proneness to shame-related negative self-evaluation (*B* = 18.034, *P* = .015) and the interaction between traumatic childbirth experience and proneness to shame-related negative self-evaluation (*B* = −0.121, *P* = .049). Thus, proneness to shame-related negative self-evaluation moderated the relationship between birth-related PTSD symptoms and post-traumatic growth in the very preterm group.

Simple slope analysis for the very preterm group revealed that even though proneness to shame-related negative self-evaluation is positively related to post-traumatic growth (as shown by the moderation model) this relationship is stronger for lower (*B* = 3.433, *P* = .013) and medium (*B* = 3.312, *P* = .012) than high proneness to shame-related negative self-evaluation (*B* = 3.192, *P* = .011). The interaction effect explained an extra 7.6% of the variance of post-traumatic growth in the model.

## Discussion

To our knowledge, this is the first study to analyze the relationship between birth-related PTSD symptoms and post-traumatic growth among women who had preterm births, as well as the effect of proneness to guilt and shame on this relationship, both generally and specifically in the postpartum context. The results of this study support the hypothesis that women who experienced preterm birth and had more severe birth-related PTSD symptoms would experience greater post-traumatic growth. The hypothesis that proneness to guilt and shame would moderate the relationship between birth-related PTSD symptoms and post-traumatic growth received only partial support. Specifically, this moderation effect was observed only in the case of proneness to shame-related negative self-evaluation, which weakened the relationship between birth-related PTSD symptoms and post-traumatic growth in women with very preterm births. Proneness to guilt, however, did not moderate this relationship and was not associated with post-traumatic growth. These results are discussed below, organized by key topics, with suggested implications for theory, practice, and policy.

### The Relationship Between Birth-Related PTSD Symptoms and Posttraumatic Growth in Women who gave Birth Prematurely

The finding that more severe birth-related PTSD symptoms were associated with greater post-traumatic growth is consistent with previous studies in different trauma contexts, which show that greater trauma exposure is linked to increased post-traumatic growth.^34,65,66^ A preterm birth is a stressful event for women and their families, with potential ongoing consequences that require coping and adaptation. If the birth is perceived as traumatic, this subjective perception, along with symptoms of post-traumatic stress, can trigger cognitive restructuring—a process where individuals reframe their thoughts and beliefs about the traumatic event.^66^ This cognitive shift, particularly when the preterm infant requires additional care and support, can lead to new perspectives. As women strive to meet their child’s needs and witness their infant’s fight for survival and growth, they may find new meaning and purpose in the experience, which can, in turn, prompt post-traumatic growth. Blix et al^65^ further contend that post-traumatic growth occurs as a response to significant life changes and adaptation to a new situation following a traumatic experience.

The association between more severe birth-related PTSD symptoms and greater post-traumatic growth in women who experienced preterm births suggests several implications. Theoretically, it reinforces the concept that trauma intensity can drive personal growth, supporting models that account for both negative and positive trauma outcomes.^67,68^ Practically, it highlights the need for trauma-informed care,^69,70^ with mental health professionals—such as psychologists and psychiatrists—providing both PTSD treatment and strategies that foster resilience and growth. For example, practioners can implement trauma-informed care by focusing on key principles such as safety, trustworthiness, collaboration, and empowerment when working with mothers who have experienced traumatic births.^71,72^ Creating a supportive and validating environment is essential for promoting both recovery and post-traumatic growth. Additionally, trauma-focused therapies like cognitive-behavioral therapy^73^ or eye movement desensitization and reprocessing,^74^ alongside strength-based approaches, can help mothers process their trauma while recognizing their capacity for resilience and growth. Moreover, as suggested by Hall et al^71^ and Mosley and Lanning,^72^ midwives, obstetricians, nurses and doulas should be trained to recognize trauma symptoms and facilitate appropriate referrals to mental health services. From a policy perspective, it underscores the importance of integrating mental health services into maternity care and ensuring resources are allocated for both PTSD treatment and growth-oriented initiatives. Future research should explore the mechanisms linking severe PTSD to post-traumatic growth, investigate various trauma responses, and evaluate the effectiveness of integrated treatment approaches. While we know that more intense trauma can sometimes foster greater growth,^34,65,66^ the specific pathways—such as emotional processing, cognitive restructuring, and social support—that lead to this growth are still unclear, as they may depend on the context of the trauma.^75^ Future studies should focus on understanding how different coping strategies or individual characteristics contribute to post-traumatic growth and examine other trauma responses beyond PTSD, such as anxiety or depression. Evaluating the effectiveness of integrated care models, which combine trauma treatment with growth-promoting interventions, is also crucial. Currently, limited research exists on how such models can be effectively applied within maternity care settings.

### The Negative Effect of Proneness to Shame on the Relationship Between Birth-Related PTSD Symptoms and Posttraumatic Growth

Previous research indicates that proneness to shame is generally maladaptive^76^ and is closely linked to feelings of failure, alienation, vulnerability, worthlessness, hopelessness, and personal inadequacy.^77^ These emotional states are inherently counterproductive to the processes that foster post-traumatic growth, particularly in the context of birth-related PTSD.

The findings support this understanding, revealing that proneness to shame-related negative self-evaluation significantly weakens the relationship between traumatic birth experiences and post-traumatic growth. Shame can trap individuals in a cycle of poor self-worth, preventing them from reframing their trauma in a way that fosters growth. This is consistent with the idea that shame, particularly when focused on self-evaluation rather than specific behaviors, exacerbates feelings of inadequacy and impedes the positive reinterpretation of trauma.^34,35^

Interestingly, current study did not find a similar moderating effect for shame withdrawal tendencies. This suggests that while withdrawal may contribute to the overall emotional burden, it is the self-critical aspect of shame—where the individual views herself as inherently flawed or unworthy—that most strongly interferes with the potential for growth after trauma. This distinction is important because it highlights that not all aspects of shame are equally detrimental to the process of post-traumatic growth. While withdrawal might lead to social isolation and a lack of support, it is the negative self-evaluation that seems to be the most significant barrier to recovery and growth. This is supported by research indicating that shame proneness is particularly damaging because it is tied closely to an individual’s sense of self-worth and identity.^34^

However, the relationship between shame and post-traumatic growth is complex. For instance, Kleim and Ehlers^53^ found that shame could, in some contexts, predict post-traumatic growth, suggesting that shame might sometimes motivate the cognitive processing needed for growth. This complexity underscores the importance of addressing the specific aspects of shame that are most harmful, particularly those related to negative self-evaluation. Shame focuses on a negative view of the self, often leading to feelings of unworthiness,^78^ which can become maladaptive when internalized, hindering growth. However, when shame is processed in an adaptive way—such as by reframing it and working through these feelings—it can motivate self-improvement and deeper reflection,^79,80^ ultimately fostering post-traumatic growth. Janoff-Bulman’s^81^ existential model of reassessment provides insight into this process, suggesting that finding meaning in traumatic experiences can mitigate the harmful effects of shame. By reframing the trauma as part of a larger life narrative, women may reduce the grip of shame and open themselves up to growth. Barr^44^ also highlights that shame is closely tied to existential experiences, and addressing it is crucial for post-traumatic growth.

In the context of birth-related trauma, women prone to shame-related negative self-evaluation might feel an exaggerated sense of responsibility for preterm birth, leading to overwhelming self-blame and reducing their potential for growth. This aligns with findings that shame can exacerbate PTSD symptoms by deepening feelings of inadequacy and isolation.^52^ When shame is internalized, it can trap women in a cycle of distress, making it difficult to engage in the cognitive processing necessary for growth. Future research should examine how different facets of shame influence the process of post-traumatic growth and explore targeted interventions that address these specific aspects.

Moreover, findings of this study reveal that when controlling for gestational age, the moderating effect of shame-related negative self-evaluation on the relationship between birth-related PTSD symptoms and post-traumatic growth diminishes. This suggests that gestational age is a significant factor influencing the psychological impact of preterm birth. Very preterm births, typically associated with greater medical complications^82^ and longer hospital stays,^83^ likely represent a more intense traumatic experience for mothers. This heightened trauma may amplify feelings of shame, particularly in those prone to negative self-evaluation, thereby making it a more significant factor in very preterm births. When gestational age is accounted for, it appears that the differences in trauma severity related to the timing of the preterm birth are what primarily drive the relationship, rather than proneness to shame-related self-evaluation alone.

Further analysis highlighted that the moderating effect of proneness to shame-related negative self-evaluation was significant only in the very preterm group, not in the moderate and late preterm group. This aligns with research suggesting that the more severe and unpredictable the birth experience, the greater the psychological burden on the mother.^56^ Very preterm births often involve intense medical interventions and a prolonged sense of helplessness, which could exacerbate a mother’s feelings of inadequacy and failure.^84^ For mothers who already have a tendency toward negative self-evaluation, this context may intensify shame, making it a critical factor that interferes with their ability to process the trauma constructively and achieve post-traumatic growth. In contrast, moderate and late preterm births, while still challenging, may not evoke the same level of trauma and perceived personal failure, thereby diminishing the impact of proneness to shame-related negative self-evaluation on post-traumatic outcomes.

In practice, these findings highlight the need for tailored psychological interventions for women who have had very preterm births. These interventions should focus on reframing shame, promoting self-compassion, and reducing internalized negative self-perceptions to transform maladaptive shame into a process that fosters healing and growth. Evidence suggests that compassion-focused therapy can help counteract self-judgment linked to shame,^85,86^ while cognitive-behavioral therapy (CBT) can challenge and reframe negative thought patterns, including those related to shame and self-evaluation, and has shown promise in PTSD treatment.^87^ Mindfulness-based practices, which encourage non-judgmental awareness of emotions, can also reduce the intensity of shame-related self-criticism.^88,89^ Future research should assess the effectiveness of these interventions in reducing shame-related self-evaluation in women who have had preterm births.

The varying impact of shame between very preterm and late preterm groups suggests that support should be adjusted based on gestational age, with more intensive care for those delivering earlier. Timely intervention is crucial, and healthcare professionals should be trained to recognize and address maladaptive shame tendencies in these women.

From a policy perspective, culturally sensitive programs are needed to address the emotional challenges of shame in mothers of preterm infants, especially those with very early births. Training healthcare providers to recognize and treat shame-related distress and advocating for early psychological interventions could improve mental health outcomes and foster post-traumatic growth in this vulnerable population.

### Why Proneness to Guilt Does not Play a Role in Posttraumatic Growth Following Birth-Related PTSD?

The findings showed no association between proneness to guilt (negative behavior evaluation or repair action tendencies) and post-traumatic growth, nor did proneness to guilt moderate the relationship between birth-related PTSD symptoms and post-traumatic growth. This contrasts with some previous studies, which found that proneness to guilt is related to post-traumatic growth^90^ and contradicts certain theoretical models. For example, the Process Model of Self-Conscious Emotions^91^ suggests that proneness to guilt encourages individuals to express regret and actively repair damage, potentially leading to post-traumatic growth. However, in the context of birth-related trauma, where the perceived “damage” is often beyond the mother’s control, such as in preterm birth, guilt may not lead to constructive actions or growth. Furthermore, guilt in this context could become maladaptive, leading to rumination rather than positive change, especially if the individual feels powerless to make effective repairs. Additionally, some scholars suggest that the role of guilt in post-traumatic growth may be mediated by adaptive stress-coping mechanisms,^92^ which might not always be activated in the context of birth-related trauma, explaining the lack of a direct association in the presented findings.

The findings highlight the need for trauma and post-traumatic growth models to account for specific emotional factors like shame, and to differentiate between the effects of shame and guilt. While shame appears to weaken the link between birth-related PTSD and post-traumatic growth, guilt does not, indicating that these emotions impact trauma recovery and growth through distinct pathways.

Future research should explore why guilt does not affect post-traumatic growth in women with preterm births, focusing on the emotional and psychological factors involved. Additionally, studies could examine whether other emotional responses or contextual factors are more relevant to post-traumatic growth in birth-related trauma.

### The Link Between Gestational Age and Birth-Related PTSD and Infant Health Status and Posttraumatic Growth

When analyzing the relationship between the main study variables and the sociodemographic and birth-related characteristics of women who had pre-term births, it was found that post-traumatic growth increases with lower gestational age, and that women whose newborns are not healthy experience more birth-related PTSD symptoms than those with healthy babies. The finding that mothers of less healthy newborns experience more birth-related PTSD symptoms underscores the substantial emotional toll that concerns about a newborn’s health can exert. Although studies that directly compare the birth-related PTSD symptoms of women with healthy versus those with health-compromised pre-term infants were not found, it is reasonable to assume that ongoing stress and anxiety related to the baby’s well-being likely intensify the trauma associated with the birth experience, leading to higher levels of PTSD symptoms. As reported by Canin and Bain^93^ these mothers may struggle with feelings of fear, helplessness, and guilt, which can exacerbate their psychological distress. Additionally, pre-term birth is often associated with more pregnancy complications^94^ and birth complications, such as operative births, which are themselves risk factors for postpartum PTSD.^95^

Although studies analyzing the relationship between gestational age and post-traumatic growth or comparing post-traumatic growth among women who had very preterm, moderate, or late preterm births were also not found, it is plausible that the increase in post-traumatic growth with lower gestational age reflects the greater challenges faced by these mothers. Mothers who encounter more severe challenges, such as earlier pre-term births, may be more likely to experience significant psychological shifts that contribute to personal growth. As they navigate the intense stress and uncertainty associated with premature childbirth, these mothers may undergo a profound reevaluation of their values, priorities, and relationships. This process can lead to enhanced resilience, a stronger sense of personal strength, and a greater appreciation for life, which are key components of post-traumatic growth.^96^ These findings highlight the potential for positive psychological outcomes even in the face of severe adversity.

Healthcare providers should recognize the heightened PTSD symptoms and potential for growth among mothers of very preterm infants or those with significant health challenges. For example, when working with mothers whose infants are in neonatal intensive care units (NICUs), providers could implement trauma-informed care practices by regularly screening for PTSD symptoms, offering psychological support, and facilitating peer support groups. These groups could help mothers process their trauma while also recognizing potential areas of personal growth, such as increased emotional resilience or stronger coping skills. Additionally, by acknowledging both the distress and the possibility of positive change, providers can help mothers feel empowered and supported in their emotional recovery.

Policies should integrate mental health services into maternity care, particularly for mothers of very preterm or health-compromised infants, ensuring adequate resources for managing PTSD and promoting post-traumatic growth. For example, maternity units could provide routine mental health screenings for PTSD, anxiety, and depression as part of postnatal care. This could be followed by offering access to on-site counseling, as well as specialized support for mothers dealing with the stress of premature birth. Additionally, policies could promote collaborative care models where mental health professionals work alongside obstetricians and pediatricians, ensuring that psychological support is seamlessly integrated into the overall healthcare experience for these mothers.

Future research should investigate the mechanisms linking gestational age and infant health with PTSD and post-traumatic growth, focusing on how severe birth experiences influence psychological outcomes and identifying effective support strategies.

### The Possible Effect of the COVID-19 Pandemic on the Given Results

It is important to note that this study was conducted during the COVID-19 pandemic, a time when women were subject to stringent safety measures aimed at preventing disease transmission and limiting social contact in birth facilities.^97^ Research has shown that specific COVID-related perinatal healthcare changes, such as visitor restrictions and mask mandates, significantly impacted the development of trauma symptoms, including PTSD, following childbirth.^98^ Despite these findings, Capatina et al^99^ did not observe an increase in reports of negative birth experiences or postnatal mental health issues related to COVID-19.

In addition, Babu et al^100^ found that while the prevalence of posttraumatic growth was similar regardless of whether childbirth occurred during or before the pandemic, there were notable differences in the relationships between childbirth-related acute stress and post-traumatic growth. Specifically, women who gave birth during the pandemic experienced a stronger association between higher levels of acute stress and increased post-traumatic growth. This relationship was not significant in the group who gave birth before the pandemic. Furthermore, elevated post-traumatic growth was linked to a reduction in childbirth-related PTSD symptoms among women who gave birth during COVID-19, a pattern not seen in those who gave birth before the pandemic.

These findings suggest that the context of the pandemic may have influenced the dynamics between birth-related PTSD symptoms and posttraumatic growth. Future research should explore these relationships further, particularly focusing on how guilt and shame proneness may affect the connection between birth related PTDS symptoms and posttraumatic growth in non-pandemic context.

### Limitations

Before drawing conclusions, it is important to consider this study’s limitations. First, the study was cross-sectional and could not establish causality, although it found a relationship between birth-related PTSD symptoms and post-traumatic growth. The sample size and number of free parameters made the data suitable for moderated regression in the overall sample. However, for the very preterm birth group, the sample size was only sufficient. The small convenience sample limits generalizability, so future research should test whether these results replicate in larger samples. Also, with a large sample comes the opportunity to do analysis using structural equation modelling, which offers several advantages over moderated regression, such as the ability to analyze multiple relationships simultaneously, handle latent variables, assess model fit, and account for measurement error, providing more accurate and comprehensive insights. Secondly, the sample was self-selected, had a high proportion of highly educated participants, and the mean score for birth-related post-traumatic stress symptoms was low. It is therefore possible that if the range and variance in birth-related post-traumatic stress symptoms in the sample were greater, analysis of moderating effects, interactions and relationships among study constructs would be more robust. Infant health, as a birth-related characteristic, was assessed based on the mother’s subjective reporting, which may represent another limitation of this study. Finally, the measure used to assess proneness to shame and guilt had four subscales (two for each construct). The advantage of this is that proneness to shame-related negative evaluations could be examined separately; however, it is also possible that measuring proneness to guilt as a whole and proneness to shame as a whole might yield different results. Similarly, the measure used presents everyday embarrassing scenario or transgressions and respondents are asked to indicate how likely it is that their experience would match the ones described. It is possible that respondents might relate better to and respond more accurately if the scenarios in the measure were focused on childbirth or the postpartum period. Additionally, the Post-traumatic growth (PTG) scale, used in this study was not highly specific to the context of premature birth. Future research should consider developing a more specific measure of proneness to guilt and shame in the perinatal period and possibly adapting the Post-traumatic growth scale for preterm birth situations.

### Conclusion

Birth-related post-traumatic stress is related not only to negative consequences but also to positive outcomes of more post-traumatic growth. This study found a relationship between proneness to shame-related negative evaluations of self and post-traumatic growth; and that shame-related negative self-evaluation moderate the relationship between birth-related PTSD symptoms and post-traumatic growth, particularly in women who have a very preterm birth. Women who experience a very preterm birth and who have high proneness to shame-related negative evaluation may need more attention from health care professionals as they could be more affected by a birth-related traumatic experience, and less likely to experience post-traumatic growth. Further research is needed to examine this further in larger, more representative samples, as well as samples with greater levels of birth-related PTSD symptoms.

## Supplemental Material

sj-docx-1-inq-10.1177_00469580241299604 – Supplemental material for Guilt-and Shame-Proneness, Birth-related Post-traumatic Stress and Post-Traumatic Growth in Women with Preterm Birth Supplemental material, sj-docx-1-inq-10.1177_00469580241299604 for Guilt-and Shame-Proneness, Birth-related Post-traumatic Stress and Post-Traumatic Growth in Women with Preterm Birth by Gabija Jarašiūnaitė-Fedosejeva, Jovita Kniežaitė, Ernesta Sakalauskienė, Susan Ayers, Annick Bogaerts and Olga Riklikienė in INQUIRY: The Journal of Health Care Organization, Provision, and Financing

sj-docx-2-inq-10.1177_00469580241299604 – Supplemental material for Guilt-and Shame-Proneness, Birth-related Post-traumatic Stress and Post-Traumatic Growth in Women with Preterm Birth Supplemental material, sj-docx-2-inq-10.1177_00469580241299604 for Guilt-and Shame-Proneness, Birth-related Post-traumatic Stress and Post-Traumatic Growth in Women with Preterm Birth by Gabija Jarašiūnaitė-Fedosejeva, Jovita Kniežaitė, Ernesta Sakalauskienė, Susan Ayers, Annick Bogaerts and Olga Riklikienė in INQUIRY: The Journal of Health Care Organization, Provision, and Financing
