# Supplementary material for: Guilt-and Shame-Proneness, Birth-related Post-traumatic Stress and Post-Traumatic Growth in Women with Preterm Birth
Source: Inquiry. 2024 Dec 2;61:00469580241299604. doi: 10.1177/00469580241299604 (PMC11613246; doi:10.1177/00469580241299604)
Supplement: sj-docx-1-inq-10.1177_00469580241299604 – Supplemental material for Guilt-and Shame-Proneness, Birth-related Post-traumatic Stress and Post-Traumatic Growth in Women with Preterm Birth [file sj-docx-1-inq-10.1177_00469580241299604.docx]

**Supplementary material**

**Table S1. Distribution of respondents sociodemographic and birth-related characteristics**

| **Sociodemographich and birth-related characterictics** | | N | % |
| --- | --- | --- | --- |
| Education | Primary school | 2 | 2.5 |
|  | Secondary school | 9 | 11.4 |
|  | Vocational school | 12 | 15.2 |
|  | Higher education | 56 | 70.9 |
| Relationship status | Single | 3 | 3.8 |
|  | Married | 54 | 68.4 |
|  | Living with a partner (cohabtation) | 21 | 26.6 |
|  | Divorced | 1 | 1.3 |
| Subjectively perceived newborn health | Healthy | 59 | 74.7 |
|  | Not healthy (has prematurity related health issues) | 15 | 19.0 |
|  | No answer | 5 | 6.3 |
