# Supplementary material for: Guilt-and Shame-Proneness, Birth-related Post-traumatic Stress and Post-Traumatic Growth in Women with Preterm Birth
Source: Inquiry. 2024 Dec 2;61:00469580241299604. doi: 10.1177/00469580241299604 (PMC11613246; doi:10.1177/00469580241299604)
Supplement: sj-docx-2-inq-10.1177_00469580241299604 – Supplemental material for Guilt-and Shame-Proneness, Birth-related Post-traumatic Stress and Post-Traumatic Growth in Women with Preterm Birth [file sj-docx-2-inq-10.1177_00469580241299604.docx]

**Supplementary material**

**Table S2. Distribution of respondents sociodemographic and birth-related characteristics**

|  | Min | Max | Mean ± SD |
| --- | --- | --- | --- |
| Age (in years) | 21 | 44 | 31.42 (5.217) |
| Gestational age (in weeks) | 22 | 36 | 31.44 (3.977) |
| Period after birth (in months) | 2 | 14 | 5.95 (3.82) |
| Number of previous childbirths | 1 | 7 | 1.84 (1.006) |
| Number of children | 1 | 8 | 1.90 (1.116) |
